# Impact of Doing Good on the Aging Self: Growth and Change Themes in Narratives of Prosocial Behavior

**DOI:** 10.1093/geroni/igaa057.1497

**Published:** 2020-12-16

**Authors:** Laura Graham, Jeanne Nakamura, Noah Ringler

**Affiliations:** 1 University of California, Riverside, Santa Monica, California, United States; 2 Claremont Graduate University, Claremont, California, United States

## Abstract

Contemporary theories consider development to be lifelong, suggesting a potential for personal growth in older adulthood. Narrative studies have found benefits of having growth themes in older adults’ broad life stories, yet there is limited research focusing on the specific experiences in later life that elicit growth. One potential for personal growth during older age is through prosocial behavior, but studies have overlooked how older adults narrate such experiences and the perceived impact the narratives have on the self. We conducted interviews with a sample of 47 older adults engaged in prosocial commitments to examine the types of perceived self-change as well as patterns in high points and low points of the experience. Narrative analyses revealed the majority of participants reported a change in the self, and half of the sample used growth themes to characterize the self-change. Inductive coding of change narratives revealed an emergent category of virtuous change (e.g., transcendence, wisdom, humanity), as well as a variety of other change categories (e.g., competence, cognitive abilities, negative changes). Those who grew narrated high points and low points with more integration than those who did not, displaying a multifaceted, complex understanding of significant episodes through a blend of both positive and negative elements within one story. These findings suggest that an integrated understanding of prosocial experiences may provide an avenue toward personal growth. Implications for research and practice are discussed.

